# Freezing Tolerance of Thermophilic Bacterial Endospores in Marine Sediments

**DOI:** 10.3389/fmicb.2019.00945

**Published:** 2019-05-03

**Authors:** Margaret A. Cramm, Anirban Chakraborty, Carmen Li, S. Emil Ruff, Bo Barker Jørgensen, Casey R. J. Hubert

**Affiliations:** ^1^Geomicrobiology Group, Department of Biological Sciences, University of Calgary, Calgary, AB, Canada; ^2^Energy Bioengineering Group, Department of Geoscience, University of Calgary, Calgary, AB, Canada; ^3^Center for Geomicrobiology, Department of Bioscience, Aarhus University, Aarhus, Denmark

**Keywords:** thermophiles, endospores, microbial ecology, extremophiles, microbial dispersal, panspermia, frozen environments, spores

## Abstract

Dormant endospores of anaerobic, thermophilic bacteria found in cold marine sediments offer a useful model for studying microbial biogeography, dispersal, and survival. The dormant endospore phenotype confers resistance to unfavorable environmental conditions, allowing dispersal to be isolated and studied independently of other factors such as environmental selection. To study the resilience of thermospores to conditions relevant for survival in extreme cold conditions, their viability following different freezing treatments was tested. Marine sediment was frozen at either −80°C or −20°C for 10 days prior to pasteurization and incubation at +50°C for 21 days to assess thermospore viability. Sulfate reduction commenced at +50°C following both freezing pretreatments indicating persistence of thermophilic endospores of sulfate-reducing bacteria. The onset of sulfate reduction at +50°C was delayed in −80°C pretreated microcosms, which exhibited more variability between triplicates, compared to −20°C pretreated microcosms and parallel controls that were not frozen in advance. Microbial communities were evaluated by 16S rRNA gene amplicon sequencing, revealing an increase in the relative sequence abundance of thermophilic endospore-forming *Firmicutes* in all microcosms. Different freezing pretreatments (−80°C and −20°C) did not appreciably influence the shift in overall bacterial community composition that occurred during the +50°C incubations. Communities that had been frozen prior to +50°C incubation showed an increase in the relative sequence abundance of operational taxonomic units (OTUs) affiliated with the class *Bacilli*, relative to unfrozen controls. These results show that freezing impacts but does not obliterate thermospore populations and their ability to germinate and grow under appropriate conditions. Indeed the majority of the thermospore OTUs detected in this study (21 of 22) could be observed following one or both freezing treatments. These results are important for assessing thermospore viability in frozen samples and following cold exposure such as the very low temperatures that would be encountered during panspermia.

## Introduction

Thermophilic endospore-forming bacteria (thermospores) have been discovered in cold marine sediments through conducting high-temperature (50°C) incubation experiments (Hubert et al., 2009; de Rezende et al., 2013; Müller et al., 2014; Volpi et al., 2017; Bell et al., 2018; Chakraborty et al., 2018; Hanson et al., 2019). These misplaced thermophiles are members of the dormant microbial seed bank and are conspicuously alien to these cold environments where they cannot grow and divide. By existing in a dormant state at temperatures below their growth and activity range they can be passively dispersed through hostile environments without suffering adverse effects. Because thermospores must originate in warm environments, their presence in cold sediments imply mechanisms of passive dispersal distribute thermospores making them unique models for studying microbial biogeography. Petroleum reservoirs and oceanic spreading centers associated with geofluid flow have been proposed as warm source environments for thermospores found in cold marine sediment (Hubert et al., 2009; Chakraborty et al., 2018).

The rate at which thermospores are dispersed is considerable. Hubert et al. (2009) found that thermospores are supplied to Arctic marine sediment at a rate of 10^8^ m^−2^ y^−1^. Many studies have observed intriguingly high numbers of thermospores in cold or mild environments (Bartholomew and Paik, 1966; Fields and Chen Lee, 1974; Marchant et al., 2002, 2008; Rahman et al., 2004; de Rezende et al., 2013; Volpi et al., 2017; Bell et al., 2018). These apparently paradoxical observations, and the fact that warm environments hospitable to thermospore germination and growth are limited, point to thermospores being well adapted for dispersal and survival (Zeigler, 2014).

Understanding the survival limits of thermospores is important if they are to be used as model organisms for studying survival in frozen ecosystems, passive dispersal on Earth, or between Earth and other planets in the context of panspermia – the theory that life is dispersed throughout the universe by vectors, including but not limited to comets, meteors, or spacecraft. While the heat tolerance of endospores is well known (Nicholson et al., 2000; Setlow, 2006; O’Sullivan et al., 2015), fewer studies have investigated the ability of endospores to withstand low temperatures including freezing conditions (Fairhead et al., 1994). Whereas sporulation at warmer temperatures results in more heat-resistant endospores (Melly et al., 2002; O’Sullivan et al., 2015), it is unclear whether endospores of thermophiles are able to survive very low sub-zero temperatures. Although thermophilic endospores are unlikely to ever encounter extreme temperatures such as −80°C on Earth, the low temperature tolerance of thermospores is interesting for several reasons. Due to their dormancy and resistance to radiation, temperature, and pressure extremes, endospore-forming bacteria have been used to study interstellar transport of microbial life by meteors or other ancient dispersal vectors (Fajardo-Cavazos et al., 2007; Nicholson, 2009).

Endospores have been shown to survive stresses associated with the three main stages of lithopanspermia, (i.e., panspermia where a rock, such as a meteorite, is the life-carrying vector). The stages are ejection from the donor planet, travel through space, and capture by the recipient planet. *Bacillus subtilis* endospores were shown to remain viable (40–100%) following simulations of the velocity, acceleration, and jerk forces encountered during impact-ejection from Mars (Mastrapa et al., 2001). Endospores of both mesophilic *B. subtilis* and thermophilic *Thermoanaerobacter siderophilus* survived entry through the Earth’s atmosphere on artificial meteorites (Fajardo-Cavazos et al., 2005; Slobodkin et al., 2015), and *B. subtilis* endospores survived the extremely high deceleration of impact onto Earth coming out of orbit (Barney et al., 2016). Horneck et al. (2008) showed that *Bacillus* endospores survive temperatures and pressures experienced by Martian meteorites found on earth as well as the stresses experienced by a trip from Earth to Mars (Horneck et al., 2012).

Surviving the stresses of ejection from a donor planet and capture by a recipient planet is not enough; panspermia also demands that endospores survive the harsh conditions of space over timescales allowing for the transit between the donor planet and a recipient planet. Irradiation is likely the harshest biocidal factor spores engaged in panspermia experience (Nicholson et al., 2005; Horneck et al., 2012) and while endospores are more resistant to UV radiation than their vegetative counterparts (Nicholson et al., 2000; Riesenman and Nicholson, 2000; Setlow, 2001, 2006), they are quickly inactivated by direct exposure to UV (Schuerger et al., 2003; Horneck et al., 2012; Panitz et al., 2015; Khodadad et al., 2017). Yet several studies showed that with minor shelter from UV endospores maintain viability (Horneck et al., 2012; Moeller et al., 2012; Vaishampayan et al., 2012) and, under protection from UV, tolerance of other stresses of the space environment, such as extremely low temperatures, may determine endospore survival. Interstellar particle temperature is ten degrees Kelvin (i.e., −263°C). While mesophilic *B. subtilis* and *B. pumilus* endospores have been used to study viability at low temperature, in vacuum pressures, and in the intense UV environment of space (Weber and Greenberg, 1985; Horneck, 1993; Nicholson et al., 2000; Horneck et al., 2012; Vaishampayan et al., 2012; Panitz et al., 2015; Khodadad et al., 2017), less is known about the survival of thermophiles.

Based on thermal inactivation kinetics, Nicholson (2003) suggested that thermophilic endospores are more likely than their mesophilic relatives to survive dormancy on panspermia-relevant timescales. Thermospores have only recently been studied with regards to the maintenance of viability during exposure to the conditions encountered during panspermia (Slobodkin et al., 2015). We therefore investigated whether thermospores survive exposure to different freezing temperatures. For this investigation we used marine sediment from an Arctic fjord of Svalbard known to contain high concentrations of thermospores (Hubert et al., 2009). We tested the hypothesis that thermospores remain viable after freezing at temperatures as low as −80°C, and that different temperature pretreatments furthermore select for a greater diversity of germinating endospores during subsequent incubation at high temperature permissive to thermophile germination and growth.

## Materials and Methods

### Freezing Pretreatment

Marine surface sediment from Smeerenburgfjorden, Svalbard, (79°42.82′ N 11°05.19′ E), previously determined to harbor thermophilic endospore-forming sulfate-reducing bacteria (Hubert et al., 2009), was used in this study. The year-round *in situ* temperature in this sediment is close to 0°C. Sediment was sampled in the summer of 2007 and stored in anoxic plastic bags at +4°C until it was used for these experiments. Wet sediment (15 g) was added to 120 mL serum bottles that were stoppered and flushed with N_2_/CO_2_ (90:10%) gas to ensure anoxic conditions. Anoxic bottles containing only sediment were frozen at either −20°C or −80°C for 10 days. The minimum temperature a cell is exposed to and the rate at which it freezes have been shown to be the factors causing the most injury to a frozen cell (Mazur and Schmidt, 1968). Both factors are accounted for in these 10-day freezing pretreatments. A parallel set of microcosms remained in a +4°C cold room during the pretreatment period and served as unfrozen positive controls.

### High-Temperature Incubation

After the freezing pretreatments, 30 mL of artificial seawater medium (Isaksen et al., 1994) amended with sulfate (20 mM), ethanol (1 mM), and six organic acids, i.e., formate, lactate, acetate, succinate, propionate, and butyrate (each to a final concentration of 1 mM), was added to each of the microcosm bottles using a syringe flushed with N_2_/CO_2_ gas. All microcosms were again flushed with N_2_/CO_2_ gas and then pasteurized at +80°C for 1 h. Immediately following pasteurization, microcosms were incubated at +50°C for 21 days to promote germination and growth of thermophilic endospore-forming bacteria.

Triplicate microcosms were prepared for each of the experimental conditions. Triplicates were subsampled immediately before and after pasteurization and then daily for the first 7 days at +50°C, and then at 10, 14, and 21 days of incubation. Subsampled aliquots were centrifuged at 14,800 rpm for 5 min to separate supernatant and pellet fractions, that were both stored at −20°C until further analysis.

### Sulfate and Organic Acid Measurement

Sulfate and organic acid concentrations were measured in supernatant subsamples at various time points during the incubation to monitor activity of thermophilic populations in the microcosms. Sulfate concentrations were determined in a Dionex ICS-5000 reagent-free ion chromatography system (Thermo Scientific) equipped with an anion-exchange column (Dionex IonPac AS22; 4 × 250 mm; Thermo Scientific), and EGC-500 K_2_CO_3_ eluent generator cartridge and a conductivity detector. The mobile phase consisted of 4.5 mM K_2_CO_3_ and 1.4 mM KHCO_3_ and was passed through the column at a constant flow rate of 1.3 mL min^−1^ while maintaining column temperature of 30°C. The sulfate detection limit was 100 μM. Organic acid concentrations were measured in an UltiMate 3000 RSLC ultra-high performance liquid chromatography system (Thermo Scientific) with a 5 mM H_2_SO_4_ mobile phase at a flow rate of 0.6 mL min^−1^ and a temperature of 60°C using an Aminex HPX-87H column (5 μm, 7.8 × 300 mm, Bio Rad). The organic acid detection limit was 2.5 μM.

### DNA Extraction and 16S rRNA Gene Amplicon Sequencing

DNA was extracted from the subsample pellets (0.3 g) using the DNeasy PowerSoil Kit (Qiagen) (formerly the PowerSoil DNA Isolation Kit, MoBio) as per the manufacturer’s protocol with the addition of a 70°C incubation for 10 min prior to bead beating as per the manufacturer’s troubleshooting guide. DNA was extracted directly from the sediment following pretreatment at −80°C or −20°C, and from the +4°C unfrozen control (i.e., prior to pasteurization), from slurry subsamples before the +50°C incubation (i.e., immediately after pasteurization) and again after 7 days of incubation at +50°C. Assessing the community composition after 7 days is consistent with observations that this is a sufficient time frame for uncovering thermospore richness in heated sediment incubations (Chakraborty et al., 2018; Hanson et al., 2019). Procedural blank DNA extractions, i.e., without any subsample added, were performed in parallel with each batch of DNA extractions. Subsequent PCR stages were performed on these blank DNA extractions to confirm the absence of contaminating DNA sequences due to the DNA extraction process.

A 427 bp fragment of the V3–V4 hypervariable region of the 16S rRNA gene was amplified using the primer pair S-D-Bact-0341-a-S-17 and S-D-Bact-0785-a-A21 (Klindworth et al., 2013). To minimize PCR bias, triplicate 25 μl PCR reactions were performed using 2 × KAPA HiFi Hot Start Ready Mix (KAPA Biosystems), a final concentration of 0.1 mM of each primer, 4–10 ng template DNA, and sterile nuclease-free water and then pooled. Touchdown PCR conditions were as follows: an initial denaturation at 95°C for 5 min, then 10 touchdown cycles of denaturation at 95°C for 30 s, a decreasing annealing temperature at 60°C to 51°C for 45 s, and extension at 72°C for 1 min. The touchdown sequence started 60°C, rather than 65°C as would be done in a classical PCR touchdown protocol, to minimize preferential amplification of high G+C sequences. After the 10 touchdown cycles, 20 additional cycles with denaturation at 95°C for 30 s, annealing at 55°C, and extension at 72°C for 1 min were performed, for a total of 30 cycles, prior to a final extension at 72°C for 5 min. Amplified 16S rRNA gene fragments 427 bp in length were prepared for sequencing as per Dong et al. (2017) and sequenced on a MiSeq Benchtop DNA sequencer (Illumina) resulting in an average library size of 44,937 reads after quality filtering. DNA extraction negatives were performed using only the buffer solutions provided for the DNA extraction protocol. PCR of the DNA extraction negatives following the same PCR conditions outlined above confirmed the absence of contamination introduced during the extraction process and these samples were not sequenced.

### Community Analysis

Community analysis was performed using the MetaAmp pipeline (Dong et al., 2017). Sequencing reads were clustered into operational taxonomic units (OTUs) using a 97% sequence identity threshold. Representative sequences for each OTU were chosen based on the UPARSE-OTU algorithm and were used for assigning taxonomy using the SILVA (version 132) database (Pruesse et al., 2012). Paired-end merging options for the MetaAmp program were 100 bp for the minimum length of overlap, and 8 as the maximum number of mismatches in the overlap region. Quality filtering allowed a maximum of 1 mismatch per primer sequence, and the maximum number of expected errors was 1. The length of the amplicon was trimmed to 350 bp. Amplicon sequences can be found in the NCBI Sequence Read Archive under accession PRJNA496528.

Operational taxonomic unit tables generated by MetaAmp (version 2.0) were used to calculate Bray–Curtis dissimilarity matrices in the R software environment (R Core Team, 2013) using a community analysis workflow based on the ‘vegan’ version 2.5–3 (Oksanen et al., 2016) and ‘cluster’ version 2.0.6 (Maechler et al., 2018) packages and custom R scripts (Ruff et al., 2019). The Bray–Curtis algorithm was chosen because it considers OTU presence/absence as well as OTU abundance, giving relatively more weight to OTUs with higher relative sequence abundance. This is especially important when a few populations dominate the communities, as is the case in thermospore enrichment experiments (Müller et al., 2014; Chakraborty et al., 2018). Microbial community similarity was visualized using non-metric multidimensional scaling (NMDS) based on dissimilarity matrices. The significance of the variance within the NMDS ordinated groups was tested using Analysis of Similarity (ANOSIM).

High-temperature (+50°C) germination experiments were required to detect viable thermospore OTUs in this study. OTUs were identified as thermospores and considered for further analyses based on the following criteria: OTUs had to be present in at least one post-incubation (day 7) sample in greater than 0.5% relative sequence abundance. Furthermore, the percent relative abundance of these OTUs had to increase by at least a factor of 10 relative to the corresponding pre-incubation library (i.e., after freezing and pasteurization, but before +50°C incubation). These criteria limited analysis only to OTUs that showed substantial increases in relative abundance. The significance of OTU relative sequence abundance between two subsampling intervals was confirmed using the STAMP application (Parks et al., 2014) using a two-sided Fisher’s Exact test, which is preferred for its accuracy with small counts (Parks and Beiko, 2010), and the Bonferroni multiple test correction to prevent false positives, resulting in a *p*-value of <0.001.

Phylogenetic analysis of OTUs was performed using the ARB software environment (Ludwig et al., 2004). Sequences included in the annotated phylogenetic tree are those of the closest cultured relatives as well as representatives of the closest uncultured relatives, in addition to the thermospore OTU sequences. Thermospore OTU representative sequences generated from MetaAmp, as well as their closest relatives (determined by BLASTn searching; Johnson et al., 2008) were aligned using the SINA aligner (Pruesse et al., 2012) and imported into the ARB-SILVA SSU Ref NR 99 132 database (Quast et al., 2013). A phylogenetic tree was calculated in ARB (Ludwig et al., 2004) using the maximum likelihood (phyML) algorithm using near-full-length (>1,300 bp) 16S rRNA reference sequences of 243 bacteria, calculated based on 1,072 alignment positions using a positional variability filter. Only conserved regions with a calculated site mutation rate of less than 8.3% were considered. The topology of the tree was validated with bootstrap support (100 re-samplings). Sequences of the thermospore OTUs and their closest relatives were added to the phylogenetic tree using the ARB Parsimony function and applying the positional variability filters for bacteria along 337 alignment positions respectively. Phylogenetic trees were visualized using iTOL version 4.2.3 (Letunic and Bork, 2006).

## Results

### Sulfate Reduction and the Production and Consumption of Organic Acids in +50°C Incubations

**FIGURE 1:**
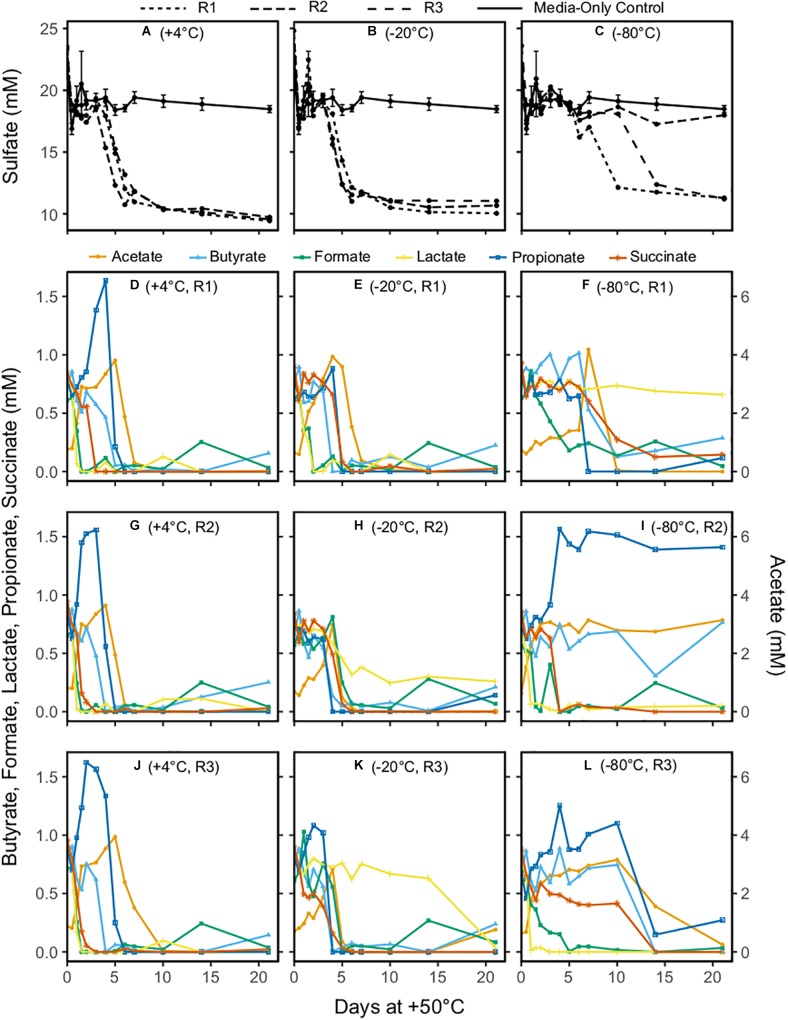
Concentrations of sulfate **(A–C)** and the organic acids acetate, butyrate, formate, lactate, propionate, and succinate **(D–L)** in sediment microcosms incubated at +50°C for 21 days. Acetate is shown on the secondary *y*-axis owing to its higher concentrations. Replicates are identified as R1, R2, and R3. In panels **(A–C)**, line types denote the medium-only control (mean of triplicates) and the individual replicates for the –20 and –80°C pretreatments and the unfrozen control. In panels **(D–L)**, symbol shapes and colors indicate the six different organic acids measured.

Patterns of net sulfate consumption in each microcosm incubated at +50°C differed depending on the freezing pretreatment. During 21 days at +50°C, the sulfate concentrations in −20°C pretreated microcosms and the +4°C unfrozen controls were similar, showing a drop in all triplicates between 3 and 6 days (Figure 1A,B). Sulfate reduction in −80°C pretreated microcosms was not observed during the first 6 days of incubation at +50°C (Figure 1C), with sulfate eventually dropping to concentrations similar to those observed in the other microcosms in two out of three replicates. In all cases where a decrease in sulfate concentration was observed, it was 7–9 mM lower than in the medium-only controls (Figure 1A–C), in agreement with the expected amount of sulfate reduction (8.75 mM) that corresponds to all organic acids being oxidized to CO_2_.

Triplicate +4°C unfrozen controls (Figure 1D,G,J) showed very similar changes in organic acid concentrations during the +50°C incubation. Formate and lactate were rapidly consumed, reaching 0 mM within 1.5 days at +50°C; it cannot be concluded with certainty whether or not this was coupled to sulfate reduction as has been observed in the early hours in similar experiments with different marine sediment (de Rezende et al., 2013, 2017). The concomitant increase in acetate during this period could be due to incomplete oxidation of lactate coupled to sulfate reduction, or acetogenesis from formate. Stoichiometric conversion of succinate to propionate was observed between day 1 and day 3 in unfrozen controls, followed by complete consumption of propionate as well as butyrate by 4–6 days. Both propionate and butyrate consumption occurred concomitantly with decreases in sulfate concentration. Acetate concentration increased by up to fourfold during the first 6 days of incubation, and then decreased to 0 mM between 6 and 10 days.

Organic acid profiles for the −20°C pretreatment showed more variability (Figure 1E,H,K) than the +4°C unfrozen controls. One replicate was similar to +4°C unfrozen control microcosms (Figure 1E) with the exception of succinate conversion to propionate (this feature was apparently less pronounced from all −20°C pretreated microcosms). The other two replicates had similar patterns to each other, with formate and lactate consumption delayed (observed after 3 days) relative to +4°C unfrozen controls. Organic acid profiles for −80°C pretreated microcosms showed the most variability between triplicates (Figure 1F,I,L), though a rapid change in organic acid concentration at +50°C was detected in some instances. Formate was consumed rapidly in one replicate (Figure 1I) and slowly in the other two (Figure 1F,L), whereas lactate was consumed rapidly in two replicates (Figure 1I,L) and was not removed at all in one replicate (Figure 1F). A threefold to fourfold increase in acetate during the first few days of incubation was observed in all three −80°C pretreated replicates. Patterns of subsequent acetate consumption in the −80°C pretreated microcosms differed from the −20°C pretreated microcosms and +4°C unfrozen controls, with either rapid, slow or no depletion (Figure 1F,I,L, respectively). In general, in all replicates following the −80°C pretreatment, changes in organic acid concentration could be observed at times when sulfate concentration was unchanging, suggesting that thermophilic sulfate reducers as well as non-sulfate-reducing thermophiles survived the freezing pretreatment and became active during +50°C incubations.

### Microbial Community Structure and Phylogeny of Thermospore OTUs

**FIGURE 2:**
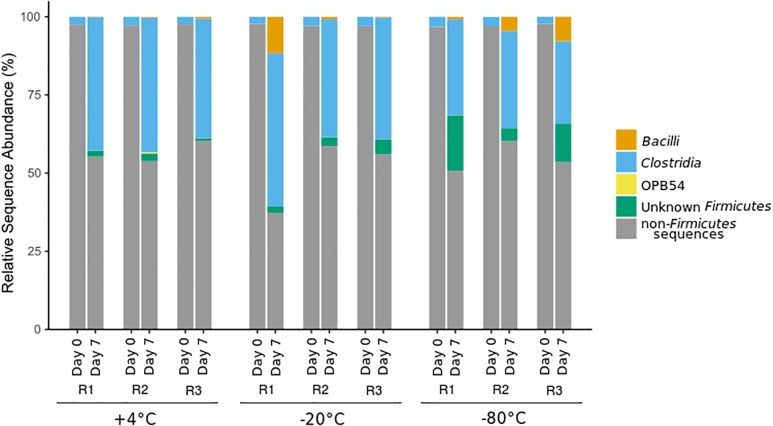
Class-level community structure within the phylum *Firmicutes* based on 16S rRNA gene amplicon sequencing before (day 0) and after (day 7) incubation at +50°C. Sequences affiliated with other phyla (not *Firmicutes*) are represented in gray. Replicates are identified as R1, R2, and R3.

An increase in the relative sequence abundance of *Firmicutes*, the phylum containing all known endospore-forming bacteria, was observed after all +50°C incubations, regardless of freezing pretreatment. *Clostridia*, the class containing all known sulfate-reducing thermospores, showed the largest increase in relative sequence abundance after +50°C incubation in all microcosms (Figure 2). Increases in the relative sequence abundance of the class *Bacilli*, and of *Firmicutes* that were unclassified at the class level, varied between replicates (R1, R2, and R3), and were most pronounced in microcosms that experienced a freezing pretreatment. *Bacilli* were 8-12% of the sequence reads in −20°C and −80°C pretreated microcosms, and <1% in +4°C pretreated unfrozen controls. In two out of three of the −80°C pretreated microcosms, the relative sequence abundance of *Firmicutes* of unknown class was >10%, whereas this category was <5% of the sequence reads in the −20°C pretreatment group, and <3% in the +4°C pretreated controls.

**FIGURE 3:**
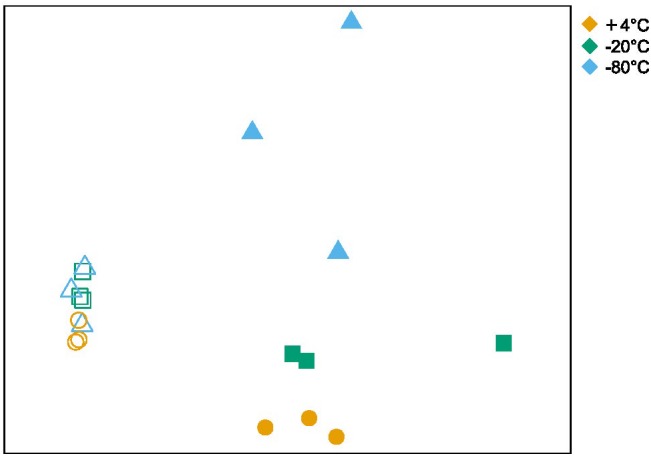
Non-metric multidimensional scaling of bacterial community similarity based on 16S rRNA gene amplicon sequencing, for samples before and after incubation at +50°C that received +4°C, –20 or –80°C freezing pretreatments. Open symbols correspond to microbial communities after pretreatment but before +50°C incubation, and closed symbols correspond to communities after 7 days of incubation at +50°C. The stress is 0.076 after 20 iterations. ANOSIM comparing the communities belonging to different pretreatment groups (after-incubation) resulted in an R statistic of 0.42 and a *p*-value of 0.011 after 999 permutations.

Alpha and beta diversity of the bacterial communities were calculated at the OTU level (clustered at 97% sequence identity) and are shown in Table 1 and Figure 3, respectively. Alpha diversity based on richness (OTU count and Chao1 Index), and evenness (Inverse Simpson Index), decreased after +50°C incubation without any notable differences in these indices between freezing pretreatments (Table 1). NMDS illustrates that the beta diversity in microcosms is significantly more variable after 7 days of incubation at +50°C compared to before the +50°C incubation (Figure 3). ANOSIM comparing the similarity between the three pretreatment groups after 7 days of incubation shows that variation in beta diversity between the groups is significant (*p* < 0.011) although the effect is relatively small (R statistic is 0.4239).

**Table 1:** Alpha diversity indices.

		Alpha Diversity Index					
		Inverse Simpson		Richness		Chao1	
Pretreatment	Days of incubation	Average	SD	Average	SD	Average	SD
+4°C	0	49.2	2.7	861.4	20.4	551.8	30.9
	7	24.6	3.7	655.8	29.1	427.0	36.4
−20°C	0	43.1	7.7	821.0	33.6	519.1	14.7
	7	16.4	1.6	579.6	115.5	350.1	75.3
−80°C	0	43.1	4.1	820.3	58.7	530.0	47.2
	7	20.4	7.6	568.9	70.8	385.7	37.0

*Values are based on the average of triplicates. Standard deviation (SD) is listed in the column to the right of the averages.*

**FIGURE 4:**
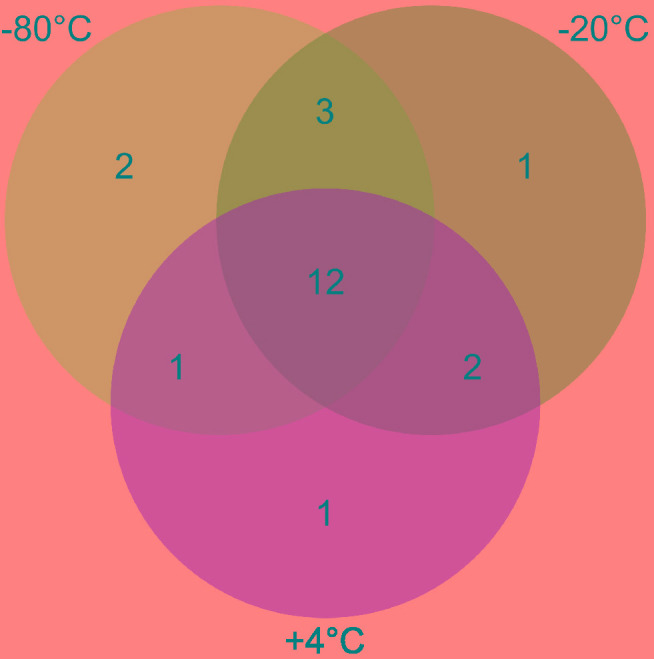
Venn diagram showing the unique and shared thermospore OTUs detected after 7 days incubation at +50°C in microcosms in each pretreatment (–80°C, –20°C, and +4°C). In total, 22 thermospore OTUs were detected in this study, out of which 12 were detected in all experimental conditions.

Twenty-two thermospore OTUs were identified from 16S rRNA gene libraries of +50°C incubations with different pretreatments. Between 8 and 16 thermospore OTUs were identified in each of the microcosms, and on average between 10 and 13 thermospore OTUs were identified within each pretreatment group (Table 2). Numbers of thermospores were not significantly different following different pretreatments based on a Kruskal-Wallis test. These OTUs accounted for 36.1–61.1% of the sequence reads for each of the microcosms after 7 days, consistent with germination subsequent and growth at +50°C by thermophiles that survived the freezing pretreatments as endospores. Of the 22 thermospore OTUs, 18 were affiliated with the class *Clostridia*, three with the class *Bacilli*, and one could not be assigned at the Class level (Table 3). The majority of identified thermospores (19 of 22) belong to spore-forming orders of *Bacilliales* within the class *Bacilli* and to spore-forming orders of *Clostridiales* within the class *Clostridia* (Table 3). A comparison of the thermospore OTUs detected following the freezing pretreatments is shown in Figure 4, revealing that 12 of the 22 thermospore OTUs were found in at least one replicate of all the pretreatment temperatures. One thermospore OTU was identified only in the +4°C unfrozen control and not in any of the −20°C or −80°C pretreated microcosms. Interestingly, six thermospore OTUs were identified only in microcosms that had been frozen (−20°C or −80°C) prior to +50°C incubation, with two of these identified only in the −80°C pretreatment group.

**Table 2:** Number of thermospore OTUs and total thermospore OTU relative sequence abundance detected after pretreatment at +4°C, −20°C, and −80°C and incubation for 7 days at +50°C.

	+4°C			−20°C			−80°C		
	R1	R2	R3	R1	R2	R3	R1	R2	R3
Number of thermospore OTUs	15	11	14	16	9	10	8	12	11
Total thermospore relative abundance (%)	42.6	43.0	37.4	61.1	39.4	42.4	47.4	36.1	44.2

*Replicates are identified as R1, R2, and R3.*

**Table 3:** Individual thermospore OTU relative sequence abundance detected after pretreatment at +4°C, −20°C, and −80°C and incubation for 7 days at +50°C.

		Relative abundance of thermospore OTU (%)								
		+4°C			−20°C			−80°C		
Thermospore #	Family	R1	R2	R3	R1	R2	R3	R1	R2	R3
Order *Bacillales* of Class *Bacilli*										

6	*Bacillaceae*				11.0				3.6	
13	Unknown	0.1		0.5	0.6	0.7	0.2	0.6	0.8	4.5
17	*Bacillaceae*									3.2

Order *Clostridiales* of Class *Clostridia*										

2	*Peptococcaceae*	11.6	12.4	12.5	12.7	20.4	21.9	8.7		
3	*Clostridiaceae*	4.2	7.4	1.2	3.7	5.5	5.4	9.1	4.9	10.8
4	*Clostridiaceae*	4.0	3.6		13.3					0.5
5	*Clostridiales Incertae Sedis*	12.1	11.1	6.7	6.1		1.2			
7	Unknown	2.6	2.3	4.2	1.7					
8	*Peptococcaceae*	0.7			2.7				8.3	
10	Cluster XI	0.3	0.5	1.1	2.7	2.8	1.6	1.9	5.1	4.5
11	Unknown	1.5		0.6					2.2	5.2
14	*Peptococcaceae*	0.2		0.4	0.5	1.5			0.3	
15	*Clostridiaceae*	0.2	0.6	0.3	0.1	0.8	0.5	0.6	0.3	0.5
16	*Clostridiaceae*						2.5			
18	*Defluviitaleaceae*	1.2	0.4	0.7	1.2	1.9	0.9	1.1	1.3	1.4
19	*Clostridiaceae*	1.9	2.2	4.9	0.2	3.1	3.4		0.5	1.3
20	*Peptostreptococcaceae*								4.7	
21	*Clostridiaceae*	0.1	0.5	0.7	1.2					0.1
22	*Clostridiaceae*			3.0						

Unknown Order of Class *Clostridia*										

9	Unknown				1.5			7.7		
12	Unknown	2.0	2.2	0.8	1.9				4.0	

Order Unknown of Class Unknown of Phylum *Firmicutes*										

1	Unknown					2.7	4.8	17.7		12.2

*Taxonomy of the thermospore OTUs are assigned at the family level. OTUs with bootstrap values <80% at the class, order, or family level are labeled as Unknown. Replicates are identified as R1, R2, and R3.*

**FIGURE 5:**
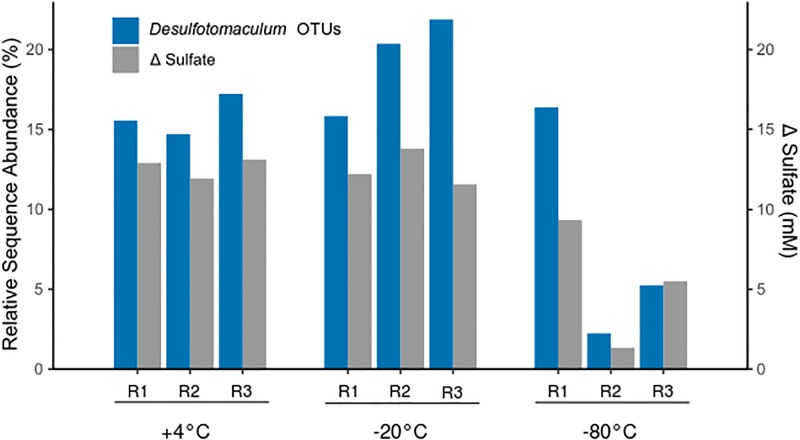
Relative sequence abundance of thermospore OTUs within the genus *Desulfotomaculum* after 7 days of incubation at +50°C (left *Y*-axis), and the corresponding amount of sulfate consumed during the first 10 days of incubation (right *Y*-axis). Replicates are identified as R1, R2, and R3. The relative sequence abundance of *Desulfotomaculum* OTUs before high-temperature incubation (i.e., day 0) was on average <0.005% in all microcosms (not shown).

Four of the 22 thermospore OTUs identified were affiliated with the genus *Desulfotomaculum*, a clade known to contain thermophilic sulfate-reducing endospore-formers. *Desulfotomaculum* thermospore OTUs were identified in all nine microcosms after 7 days of +50°C incubation. Figure 5 shows that increased relative sequence abundance of *Desulfotomaculum* thermospores corresponds with decreases in sulfate concentration at +50°C. The sulfate concentration in two of the replicates that were pretreated at −80°C drops only minimally after 10 days at +50°C (Figure 1C) compared to the other microcosms; these replicate bottles (R2, R3) have much lower levels of *Desulfotomaculum* thermospores in the corresponding amplicon libraries (Figure 5). Specifically, Thermospore 2, most closely related to *Desulfotomaculum thermosapovorans*, is identified in all microcosms that experience a rapid drop in sulfate concentration before 10 days, but not in these two microcosms. This OTU had on average the highest relative sequence abundance in the other seven microcosms (14%) pointing to this organism as being a key driver of sulfate reduction in these experiments (Table 2).

**FIGURE 6:**
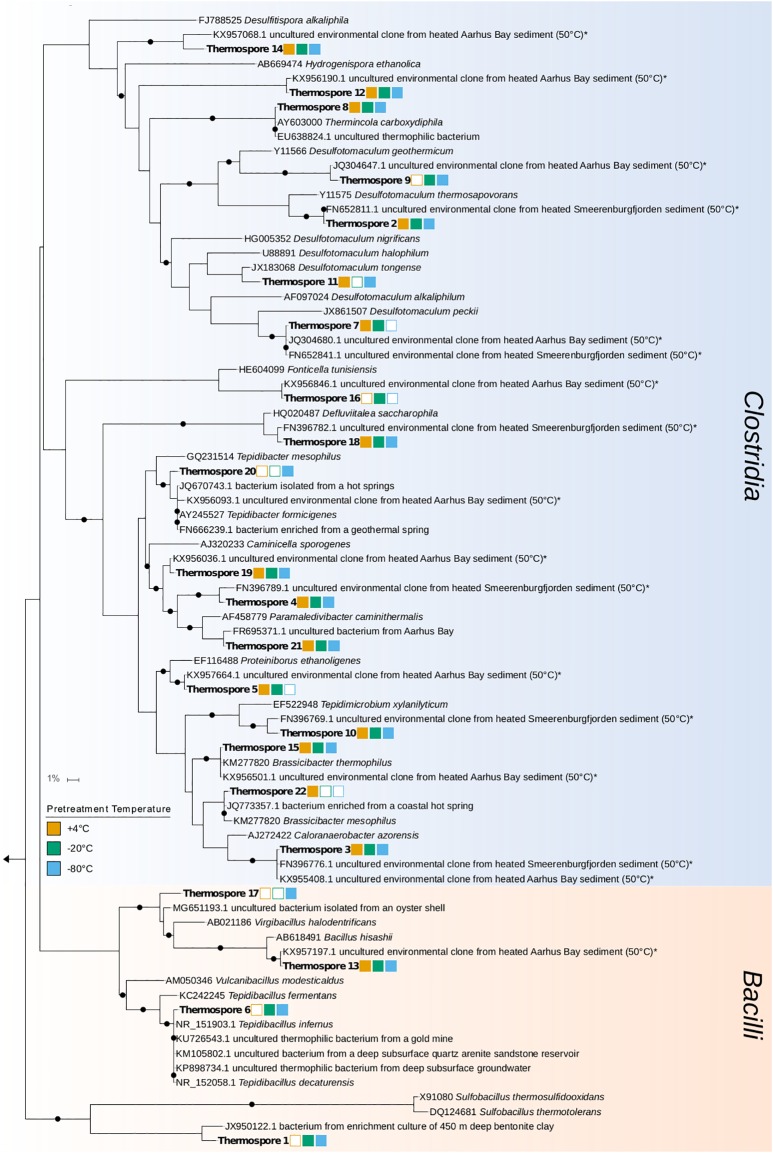
Phylogenetic tree of annotated 16S rRNA gene sequences from 22 thermospore OTUs identified in this study (in bold) and their closest cultured and uncultured relatives (determined by BLASTn searching; Johnson et al., 2008). Percent identity for uncultured relatives is between 98 and 100%, and for cultured relatives is between 87 and 100%. Uncultured relatives identified in similar sediment heating experiments are indicated by an asterisk. Bootstrap values greater than 80% after 100 re-samplings are indicated by black circles at the nodes. Squares to the right of the thermospore OTUs indicate the pretreatments prior to the +50°C incubations in which that thermospore was identified (an empty square indicates that the OTU was not identified in any replicates in that pretreatment). The scale bar indicates 1% sequence divergence as inferred by PhyML.

The thermospores identified in this study are closely related to cultured bacteria and environmental sequences from similar biogeographical studies (i.e., sediment heating experiments) as well as from warm environments inhabited by thermophiles. Of the 22 thermospores identified here, 16 are closely related to thermospores that have previously been detected in sediment heating experiments (Figure 6). Only four (thermospore OTUs 5, 7, 16, and 22) of the 22 OTUs were not identified in any of the −80°C pretreated microcosms, supporting the notion that many thermospores can be enriched from sediments frozen at temperatures as low as −80°C, potentially enabling biogeography studies using samples preserved in this way. Out of the 22 thermospores identified in this study, 16 were not identified in other thermospore studies using sediment from the same site (Hubert et al., 2009, 2010).

## Discussion

Previous studies have shown that thermospores from cold marine sediments germinate upon incubation at high temperature (Hubert et al., 2009, 2010; de Rezende et al., 2013; Müller et al., 2014; Volpi et al., 2017; Bell et al., 2018; Chakraborty et al., 2018; Hanson et al., 2019). In this study, as in previous studies, sulfate consumption at +50°C after pasteurization corresponded with an increase in the relative sequence abundance of putative sulfate-reducing bacteria. This is consistent with the survival, germination and growth of thermophilic *Desulfotomaculum* endospores. Sulfate reduction and organic acid consumption observed in the microcosms for both experimental pretreatments (−20°C, −80°C) and the +4°C unfrozen control point to thermophile activity at +50°C and the ability of different thermospores to remain viable after freezing at −20°C and −80°C. Nearly all of the thermospores identified in this study (21 out of 22 OTUs) were detected following a 10-day freezing pretreatment, with the great majority (18 out of 22) observed to increase in relative abundance after being exposed to −80°C. This suggests that the viability of these thermospores is largely unaffected by freezing, and that the low temperature tolerance of thermospores extends to −80°C. These results are in alignment with previous reports suggesting no loss in endospore viability after −20°C storage (Freeman and Wilcox, 2003; Mah et al., 2009) and extend the lower temperature limit for maintaining viability to −80°C for many endospores of thermophilic bacteria. The discovery that many of the thermospores identified here, from different pretreatment groups, share phylogenetic similarity to thermospores that have been the focus of other thermospore germination studies, indicates that frozen storage of marine sediment should not preclude their use in studies of biogeography and dispersal that rely on sediment heating to germinate thermospores.

While bacterial activity was evident after freezing pretreatments (Figure 1), variability between triplicates was also common and may be driven by differences in the thermospore diversity and/or abundance of viable endospores within each individual microcosm bottle following different experimental pretreatments, or by purely stochastic effects. Differences in organic acid production and consumption were more pronounced within the −20°C and −80°C pretreatment microcosms, compared to the +4°C unfrozen controls, suggesting freezing and freezing temperature affect the number of viable thermospores. Non-uniform sulfate depletion among triplicates following the −80°C pretreatment, and the later onset of sulfate reduction in these incubations (compared to the −20°C and +4°C pretreatments) suggests that some sulfate reducers may be present in low abundance or have reduced viability following freezing at −80°C. For example, thermospore OTU 2 (related to *Desulfotomaculum thermosapovorans*) was consistently detected in microcosms pretreated at −20°C or +4°C prior to heating, but not in microcosms pretreated at −80°C, suggesting that fewer viable endospores were present after the −80°C exposure. On the other hand, other thermospores that were only detected after freezing, or only detected after −80°C freezing, albeit sporadically across triplicates (thermospore OTUs 1, 6, 9, 16, 17, and 20) may simply be robust freeze-tolerant endospores that are present *in situ* in low abundance.

This latter group of thermospores indicates that freezing pretreatment is able to reveal a different complement of thermospores from a given sediment sample and thereby uncover a greater diversity of these target organisms when multiple different pretreatments are employed before high-temperature enrichment. It is possible that some thermospores experience competitive exclusion when unfrozen sediment is incubated at +50°C (i.e., this study and previous studies), and that freezing pretreatment impairs other thermospores in the sediment sample (those less tolerant to freezing) allowing the otherwise-excluded thermospores to become enriched and therefore detectable in +50°C incubations following freezing. This ability to uncover a greater diversity of organisms engaged in potential long-distance and long-term passive dispersal is valuable for biogeography studies employing the thermospore study system. The results presented here also further confirm that samples can be frozen and still used in such investigations as was done by Chakraborty et al. (2018) in an investigation of thermospores in Gulf of Mexico sediments. Given the long-term survival potential of endospores, this feature is particularly useful in instances where study design depends on compilations of samples from various different archives to address specific biogeography questions (e.g., Müller et al., 2014).

The larger representation of *Bacilli* sequences in the microcosms that were frozen (−20°C and −80°C) before +50°C incubation have not been observed in previous studies of this sediment (Hubert et al., 2009, 2010). Presumably these *Bacilli* endospores are sufficiently abundant in the sediment and are thus present initially in all microcosms, but only become detectable in the +50°C-active thermospore community after freezing renders certain *Clostridia* non-viable or otherwise impairs their germination. This suggests that these *Bacilli* are better able to tolerate freezing compared to certain *Clostridia* thermospores (e.g., thermospore OTUs 5, 7, 12, and 21 were reproducibly detected only in the +4°C unfrozen control microcosms). Genes for the sporulation process are generally conserved among spore-forming *Firmicutes* (Galperin, 2013), yet there are differences between the complement of sporulation genes possessed by different endospore-formers that may explain differential freezing tolerance. Fairhead et al. (1994) observed that the absence of small acid-soluble proteins (SASPs) contributed to a decrease in endospore viability after freeze-drying suggesting that SASPs may play an important role in endospore tolerance to low temperature stress. SASPs bind to DNA within the spore core and are well known to offer protection against radiation and dry heat (Fairhead et al., 1993; Setlow, 2001, 2007; Paredes-Sabja et al., 2008). At the class level, *Bacilli* and *Clostridia* differ notably in their SASP complement; *Bacilli* generally contain between 11 and 22 different SASP genes, whereas *Clostridia* often contain only two (Galperin et al., 2012). Meaney et al. (2016) suggest that the protections conferred by different SASPs encoded by *Clostridium botulinum* are additive (i.e., in protecting DNA against chemical damage). It is possible that the greater number of SASP genes leads to increased tolerance to freezing in certain *Bacilli* and other thermospores, including those corresponding to thermospore OTUs that were detected in higher relative sequence abundance in microcosms that were frozen prior to +50°C incubation (Table 3). Further studies into the relationship between SASP genes and thermospore freezing tolerance may shed light on genomic determinants to freezing tolerance.

## Conclusion

This is the first study exploring the freezing tolerance of bacterial endospore populations from the natural environment, and from geologic samples in particular. Furthermore, while some previous studies have explored the freezing tolerance of mesophilic endospores in pure culture (Weber and Greenberg, 1985; Fairhead et al., 1994; Jafari et al., 2016), this is the first study exploring the freezing tolerance of thermospores specifically, discovering a number of different bacteria that form spores that survive freezing conditions. Our results suggest that storage of thermospores at −20°C or −80°C does not preclude their use in biogeography investigations relying on high-temperature incubation experiments. Endospore freezing tolerance is relevant to their proposed usefulness as model organisms for studying microbial dispersal and broadens the scope of such investigations to consider capabilities of microorganisms for dispersal not only on Earth but between Earth and other planets in our solar system. For example, our results show that thermospores that survive dispersal from Earth to Mars should remain viable in soil on the surface of Mars, where the average temperature fluctuates between −10 and −76°C (Schofield et al., 1997; Horneck et al., 2012).

The lower temperature tolerance of thermophilic endospores for maintaining viability during dormancy, if one exists at all, remains unconstrained given that many thermospores were able to survive at −80°C. These may be good candidates for additional studies of tolerance to other extreme conditions. Thermospores presumably exhibit tolerances to radiation, extreme temperature, and pressure extremes similar to their mesophilic counterparts, and are projected to remain viable for much longer time scales (Nicholson, 2003). Thermospores may thus be uniquely prepared to withstand conditions required for panspermia and should be considered in studies exploring interplanetary dispersal.

## Author Contributions

BJ and CH planned and conducted the Arctic sampling expeditions. MC and CH designed the sediment freezing experiments with input from AC.MC conducted all experiments. MC and CL prepared the 16S rRNA gene amplicon libraries. MC performed the data analysis with support from SR and AC. MC and CH wrote the manuscript with input from AC, CL, SR, and BJ.

## Conflict of Interest Statement

The authors declare that the research was conducted in the absence of any commercial or financial relationships that could be construed as a potential conflict of interest.

## References

[B1] Barney B. L. Pratt S. N. Austin D. E. (2016). Survivability of bare, individual *Bacillus subtilis* spores to high-velocity surface impact: implications for microbial transfer through space. *Planet. Space Sci.* 125 20–26. 10.1016/j.pss.2016.02

[B2] Bartholomew J. W. Paik G. (1966). Isolation and identification of obligate thermophilic sporeforming Bacilli from ocean basin cores. *J. Bacteriol.* 92 635–638. 5922538 10.1128/jb.92.3.635-638.1966 PMC276302

[B3] Bell E. Blake L. I. Sherry A. Head I. A. Hubert C. R. J. (2018). Distribution of thermophilic endospores in temperate estuary indicate that dispersal history structures sediment microbial communities. *Environ. Microbiol.* 20 1134–1147. 10.1111/1462-2920.14056 29393553 PMC6849807

[B4] Chakraborty A. Ellefson E. Li C. Gittens D. Brooks J. M. Bernard B. B. (2018). Thermophilic endospores associated with migrated thermogenic hydrocarbons in deep Gulf of Mexico marine sediments. *ISME J.* 12 1895–1906. 10.1038/s41396-018-0108-y 29599524 PMC6052102

[B5] de Rezende J. R. Hubert C. R. J. Røy H. Kjeldsen K. U. Jørgensen B. B. (2017). Estimating the abundance of endospores of sulfate-reducing bacteria in environmental samples by inducing germination and exponential growth. *Geomicrobiol. J.* 34 338–345. 10.1080/01490451.2016.1190805

[B6] de Rezende J. R. Kjeldsen K. U. Hubert C. R. J. Finster K. Loy A. Jørgensen B. B. (2013). Dispersal of thermophilic *Desulfotomaculum* endospores into Baltic Sea sediments over thousands of years. *ISME J.* 7 72–84. 10.1038/ismej.2012.83 22832348 PMC3524260

[B7] Dong X. Kleiner M. Sharp C. E. Thorson E. Li C. Liu D. (2017). Fast and simple analysis of MiSeq amplicon sequencing data with MetaAmp. *Front. Microbiol.* 8:1461. 10.3389/fmicb.2017.01461 28824589 PMC5540949

[B8] Fairhead H. Setlow B. Setlow P. (1993). Prevention of DNA damage in spores and in-vitro by small, acid-soluble proteins from *Bacillus* species. *J. Bacteriol.* 175 1367–1374. 8444799 10.1128/jb.175.5.1367-1374.1993 PMC193223

[B9] Fairhead H. Setlow B. Waites W. M. Setlow P. (1994). Small, acid-soluble proteins bound to DNA protect *Bacillus subtilis* spores from being killed by freeze-drying. *Appl. Environ. Microbiol.* 60 2647–2649. 8074535 10.1128/aem.60.7.2647-2649.1994 PMC201697

[B10] Fajardo-Cavazos P. Link L. Melosh H. J. Nicholson W. L. (2005). *Bacillus subtilis* spores on artificial meteorites survive hypervelocity atmospheric entry: implications for lithopanspermia. *Astrobiology* 5 726–736. 10.1089/ast.2005.5.726 16379527

[B11] Fajardo-Cavazos P. Schuerger A. C. Nicholson W. L. (2007). Testing interplanetary transfer of bacteria between Earth and Mars as a result of natural impact phenomena and human spaceflight activities. *Acta Astronaut.* 60 534–540. 10.1016/j.actaastro.2006.09.018

[B12] Fields M. L. Chen Lee P. P. (1974). *Bacillus stearothermophilus* in soils of Iceland. *Appl. Microbiol.* 28 638–640. 4421871 10.1128/am.28.4.638-640.1974 PMC186787

[B13] Freeman J. Wilcox M. H. (2003). The effects of storage conditions on viability of *Clostridium difficile* vegetative cells and spores and toxin activity in human faeces. *J. Clin. Pathol.* 56 126–128. 10.1136/jcp.56.2.126 12560391 PMC1769877

[B14] Galperin M. (2013). Genome diversity of spore-forming *Firmicutes*. *Microbiol. Spectrum* 1:TBS-0015-2012 10.1128/microbiolspectrum.TBS-0015-2012 PMC4306282 26184964

[B200] Galperin M. Y. Mekhedov S. L. Puigbo P. Smirnov S. Wolf Y. I. Rigden D. J. (2012). Genomic determinants of sporulation in *Bacilli* and *Clostridia*: towards the minimal set of sporulation-specific genes. *Environ. Microbial.* 14 2870–2890. 10.1111/j.1462-2920.2012.02841.x 22882546 PMC3533761

[B15] Hanson C. Müller A. L. Loy A. Dona C. Appel R. Jørgensen B. B. (2019). Historical factors associated with past environments influence the biogeography of thermophilic endospores in arctic marine sediments. *Front. Microbiol.* 10:245. 10.3389/fmicb.2019.00245 30873129 PMC6403435

[B16] Horneck G. (1993). Responses of *Bacillus subtilis* spores to space environment: results from experiments in space. *Orig. Life Evol. Biosph.* 23 37–52. 10.1007/BF01581989 8433836

[B17] Horneck G. Moeller R. Cadet J. Douki T. Mancinelli R. L. Nicholson W. (2012). Resistance of bacterial endospores to outer space for planetary protection purposes – experiment PROTECT of the EXPOSE-E mission. *Astrobiology* 12 445–456. 10.1089/ast.2011.0737 22680691 PMC3371261

[B18] Horneck G. Stöffler D. Ott S. Hornemann U. Cockell C. S. Moeller R. (2008). Microbial rock inhabitants survive hypervelocity impacts on Mars-like host planets: first phase of lithopanspermia experimentally tested. *Astrobiology* 8 17–44. 10.1089/ast.2007.0134 18237257

[B19] Hubert C. Arnosti C. Brüchert V. Loy A. Vandieken V. Jørgensen B. B. (2010). Thermophilic anaerobes in Arctic marine sediments induced to mineralize complex organic matter at high temperature. *Environ. Microbiol.* 12 1089–1104. 10.1111/j.1462-2920.2010.02161.x 20192966

[B20] Hubert C. Loy A. Nickel M. Arnosti C. Baranyi C. Brüchert V. (2009). A constant flux of diverse thermophilic bacteria into the cold Arctic seabed. *Science* 325 1541–1544. 10.1126/science.1174012 19762643

[B21] Isaksen M. F. Bak F. Jørgensen B. B. (1994). Thermophilic sulfate-reducing bacteria in cold marine sediment. *FEMS Microbiol. Ecol.* 14 1–8.

[B22] Jafari M. Alebouyeh M. Mortazavian A. M. Hosseini H. Ghanati K. Amiri Z. (2016). Influence of heat shock temperatures and fast freezing on viability of probiotic sporeformers and the issue of spore plate count versus true numbers. *Nutr. Food Sci. Res.* 3 35–42. 10.18869/acadpub.nfsr.3.1.35

[B23] Johnson M. Zaretskaya I. Raytselis Y. Merezhuk Y. McGinnis S. Madden T. L. (2008). NCBI BLAST: a better web interface. *Nucleic Acids Res.* 36 W5–W9. 10.1093/nar/gkn201 18440982 PMC2447716

[B24] Khodadad C. L. Wong G. M. James L. M. Thakrar P. J. Lane M. A. Catechis J. A. (2017). Stratosphere conditions inactivate bacterial endospores from a mars spacecraft assembly facility. *Astrobiology* 17 337–350. 10.1089/ast.2016.1549 28323456 PMC5399745

[B25] Klindworth A. Pruesse E. Schweer T. Peplies J. Quast C. Horn M. (2013). Evaluation ofgeneral 16S ribosomal RNA gene PCR primers for classical and next-generation sequencing-based diversity studies. *Nucleic Acids Res.* 41:e1. 10.1093/nar/gks808 22933715 PMC3592464

[B26] Letunic I. Bork P. (2006). Interactive Tree Of Life (iTOL): an online tool for phylogenetic tree display and annotation. *Bioinformatics* 23 127–128. 10.1093/bioinformatics/btl529 17050570

[B27] Ludwig W. Strunk O. Westram R. Richter L. Meier H. Yadhukumar (2004). ARB: a software environment for sequence data. *Nucleic Acids Res.* 32 1363–1371. 10.1093/nar/gkh293 14985472 PMC390282

[B28] Maechler M. Rousseeuw P. Struyf A. Hubert M. Hornik K. (2018). *Cluster: Cluster Analysis Basics and Extensions. R Package Version 2.0.7-1*. .

[B29] Mah J.-H. Kang D.-H. Tang J. (2009). Comparison of viability and heat resistance of *Clostridium sporogenes* stored at different temperatures. *J. Food Sci.* 74 M23–M27. 10.1111/j.1750-3841.2008.00984.x 19200102

[B30] Marchant R. Banat I. M. Rahman T. J. Berzano M. (2002). The frequency and characteristics of highly thermophilic bacteria in cool soil environments. *Environ. Microbiol.* 4 595–602. 10.1046/j.1462-2920.2002.00344.x 12366754

[B31] Marchant R. Franzetti A. Pavlostathis S. G. Tas D. O. Erdbrûgger I. Ûnyayar A. (2008). Thermophilic bacteria in cool temperate soils: are they metabolically active or continually added by global atmospheric transport? *Appl. Microbiol. Biotechnol.* 78 841–852. 10.1007/s00253-008-1372-y 18256821

[B32] Mastrapa R. M. E. Glanzberg H. Head J. N. Melosh H. J. Nicholson W. L. (2001). Survival of bacteria exposed to extreme acceleration: implications for panspermia. *Earth Planet Sci. Lett.* 189 1–8.

[B33] Mazur P. Schmidt J. (1968). Interactions of cooling velocity, temperature, and warming velocity on the survival of frozen and thawed yeast. *Cryobiol* 5 1–17. 10.1016/S0011-2240(68)80138-5 5760041

[B34] Meaney C. A. Cartman S. T. McClure P. J. Minton N. P. (2016). The role of small acid-soluble proteins (SASPs) in protection of spores of *Clostridium botulinum* against nitrous acid. *Int. J. Food Micrbiol.* 216 25–30. 10.1016/j.ijfoodmicro.2015.08.024 26386202

[B35] Melly E. Genest P. C. Gilmore M. E. Little S. Popham D. L. Dirks A. (2002). Analysis of the properties of spores of *Bacillus subtilis* prepared at different temperatures. *J. Appl. Microbiol.* 92 1105–1115. 10.1046/j.1365-2672.2002.01644.x 12010551

[B36] Moeller R. Reitz G. Nicholson W. L. the Protect Team Horneck G. (2012). Mutagenesis in bacterial spores exposed to space and simulated martian conditions: data from the EXPOSE-E spaceflight experiment PROTECT. *Astrobiology* 12 457–468. 10.1089/ast.2011.0739 22680692

[B37] Müller A. L. de Rezende J. R. Hubert C. R. J. Kjeldsen K. U. Lagkouvardos I. Berry D. (2014). Endospores of thermophilic bacteria as tracers of microbial dispersal by ocean currents. *ISME J.* 8 1153–1165. 10.1038/ismej.2013.225 24351936 PMC4030223

[B38] Nicholson W. L. (2003). Using thermal inactivation kinetics to calculate the probability of extreme spore longevity: implications for paleomicrobiology and lithopanspermia. *Orig. Life Evol. Biosph.* 33 621–631. 10.1023/A:1025789032195 14601931

[B39] Nicholson W. L. (2009). Ancient micronauts: interplanetary transport of microbes by cosmic impacts. *Trends Microbiol.* 17 243–250. 10.1016/j.tim.2009.03.004 19464895

[B40] Nicholson W. L. Munakata N. Horneck G. Melosh H. J. Setlow P. (2000). Resistance of *Bacillus* endospores to extreme terrestrial and extraterrestrial environments. *Microbiol. Mol. Biol. Rev.* 64 548–572. 10.1128/MMBR.64.3.548-572.2000 10974126 PMC99004

[B41] Nicholson W. L. Schuerger A. C. Setlow P. (2005). The solar UV environment and bacterial spore UV resistance: considerations for earth-to-mars transport by natural processes and human spaceflight. *Mutat. Res.* 571 249–264. 10.1016/j.mrfmmm.2004.10.012 15748651

[B42] Oksanen J. Blanchet F. Kindt R. Legendre P. Minchin P. O’Hara R. (2016). *Vegan: Community Ecology Package. R Package Version 2.3-4*.

[B43] O’Sullivan L. A. Roussel E. G. Weightman A. J. Webster G. Hubert C. R. Bell E. (2015). Survival of *Desulfotomaculum* spores from estuarine sediments after serial autoclaving and high-temperature exposure. *ISME J.* 9 922–933. 10.1038/ismej.2014.190 25325382 PMC4817712

[B44] Panitz C. Horneck G. Rabbow E. Rettberg P. Moeller R. Cadet J. (2015). The SPORES experiment of the EXPOSE-R mission: *Bacillus subtilis* spores in artificial meteorites. *Int. J. Astrobiol.* 14 105–114. 10.1017/S1473550414000251

[B45] Paredes-Sabja D. Raju D. Torres J. A. Sarker M. R. (2008). Role of small, acid-soluble spore proteins in the resistance of *Clostridium perfringens* spores to chemicals. *Int. J. Food Microbiol.* 122 333–335. 10.1016/j.ijfoodmicro.2007.12.006 18221812

[B46] Parks D. H. Beiko R. G. (2010). Identifying biologically relevant difference between metagenomic communities. *Bioinformatics* 26 715–721. 10.1093/bioinformatics/btq041 20130030

[B47] Parks D. H. Tyson G. W. Hugenholtz P. Beiko R. G. (2014). STAMP: statistical analysis of taxonomic and functional profiles. *Bioinformatics* 30 3123–3124. 10.1093/bioinformatics/btu494 25061070 PMC4609014

[B48] Pruesse E. Peplies J. Glöckner F. O. (2012). SINA: Accurate high-throughput multiple sequence alignment of ribosomal RNA genes. *Bioinformatics* 28 1823–1829. 10.1093/bioinformatics 22556368 PMC3389763

[B49] Quast C. Pruesse E. Yilmaz P. Gerken J. Schweer T. Yarza P. (2013). The SILVA ribosomal RNA gene database project: improved data processing and web-based tools. *Nucleic Acids Res.* 41 590–596. 10.1093/nar/gks1219 23193283 PMC3531112

[B50] R Core Team (2013). *R: A Language and Environment for Statistical Computing*. Vienna: R Foundation for Statistical Computing.

[B51] Rahman T. J. Marchant R. Banat I. M. (2004). Distribution and molecular investigation of highly thermophilic bacteria associated with cool soil environments. *Biochem. Soc. Trans.* 32 209–213. 10.1042/bst0320209 15046573

[B52] Riesenman P. J. Nicholson W. L. (2000). Role of the spore coat layers in *Bacillus subtilis* spore resistance to hydrogen peroxide, artificial UV-C, UV-B, and solar UV radiation. *Appl. Environ. Microbiol.* 66 620–626. 10.1128/AEM.66.2.620-626.2000 10653726 PMC91871

[B53] Ruff S. E. Felden J. Gruber-Vodicka H. R. Marcon Y. Knittel K. Ramette A. (2019). In situ development of a methanotrophic microbiome in deep-sea sediments. *ISME J.* 13 197–213. 10.1038/s41396-018-0263-1 30154496 PMC6298960

[B54] Schofield J. T. Barnes J. R. Crisp D. Haberle R. M. Larsen S. Magalhães J. A. (1997). The mars pathfinder atmospheric structure investigation/meteorology (ASI/MET) experiment. *Science* 278 1752–1758. 10.1126/science.278.5344.1752 9388169

[B55] Schuerger A. C. Mancinelli R. L. Kern R. G. Rothschild L. J. McKay C. P. (2003). Survival of endospores of *Bacillus subtilis* on spacecraft surfaces under simulated martian environments: implications for the forward contamination of Mars. *Icarus* 165 253–276. 10.1016/S0019-1035(03)00200-8 14649627

[B56] Setlow P. (2001). Resistance of spores of *Bacillus* species to ultraviolent light. *Environ. Mol. Mutagen.* 38 97–104. 10.1002/em.1058 11746741

[B57] Setlow P. (2006). Spores of *Bacillus subtilis*: their resistance to and killing by radiation, heat and chemicals. *J. Appl. Microbiol.* 101 514–525. 10.1111/j.1365-2672.2005.02736.x 16907802

[B58] Setlow P. (2007). I will survive: DNA protection in bacterial spores. *Trends. Microbiol.* 15 172–180. 10.1016/j.tim.2007.02.004 17336071

[B59] Slobodkin A. Gavriloc S. Ionov V. Iliyin V. (2015). Spore-forming thermophilic bacterium within artificial meteorite survives entry into the Earth’s atmosphere on FOTON-M4 satellite landing module. *PLoS One* 10: e0132611. 26151136 10.1371/journal.pone.0132611 PMC4494708

[B60] Vaishampayan P. A. Rabbow E. Horneck G. Venkateswaran J. J. (2012). Survival of *Bacillus pumilus* spores for a prolonged period of time in real space conditions. *Astrobiology* 12 487–497. 10.1089/ast.2011.0738 22680694

[B61] Volpi M. Lomstein B. A. Sichert A. Røy H. Jørgensen B. B. Kjeldsen K. U. (2017). Identity, abundance, and reactivation kinetics of thermophilic fermentative endospores in cold marine sediment and seawater. *Front. Microbiol.* 8:131. 10.3389/fmicb.2017.00131 28220111 PMC5292427

[B62] Weber P. Greenberg J. M. (1985). Can spores survive in interstellar space? *Nature* 316 403–407. 10.1038/316403a0

[B63] Zeigler D. R. (2014). The Geobacillus paradox: why is a thermophilic bacterial genus so prevalent on a mesophilic planet? *Microbiology* 160 1–11. 10.1099/mic.0.071696-0 24085838

